# Perceptions of the role of general practice and practical support measures for carers of stroke survivors: a qualitative study

**DOI:** 10.1186/1471-2296-12-57

**Published:** 2011-06-23

**Authors:** Nan Greenwood, Ann Mackenzie, Ruth Harris, Will Fenton, Geoffrey Cloud

**Affiliations:** 1 Faculty of Health and Social Care Sciences, St George's University of London and Kingston University, Cranmer Terrace, London, SW17 0RE, UK; 2 Heathbridge Practice, Upper Richmond Road. London, SW15 2TL, UK; 3 Department of Neurology, St George's Hospital, Blackshaw Road, Tooting, London, SW17 0RE, UK

**Keywords:** general practice, carer, caregiver, stroke

## Abstract

**Background:**

Informal carers frequently suffer adverse consequences from caring. General practice teams are well positioned to support them. However, what carers of stroke survivors want and expect from general practice, and the practical support measures they might like, remain largely unexplored.

The aims of this study are twofold. Firstly it explores both the support stroke carers would like from general practice and their reactions to the community based support proposed in the New Deal. Secondly, perceptions of a general practice team are investigated covering similar topics to carer interviews but from their perspective.

**Methods:**

Semi-structured interviews were conducted with 13 stroke carers and 10 members of a general practice team. Carers' experiences and expectations of general practice and opinions of support measures from recent government policy were explored. General practice professionals were asked about their perceived role and their perceptions of carers' support needs. Interviews were content analysed.

**Results:**

Carers' expectations of support from general practice were low and they neither received nor expected much support for themselves. General practice was seen as reactive primarily because of time constraints. Some carers would appreciate emotional support but others did not want additional services. Responses to recent policy initiatives were mixed with carers saying these might benefit other carers but not themselves.

General practice professionals' opinions were broadly similar. They recognise carers' support needs but see their role as reactive, focussed on stroke survivors, rather than carers. Caring was recognised as challenging. Providing emotional support and referral were seen as important but identification of carers was considered difficult. Time constraints limit their support. Responses to recent policy initiatives were positive.

**Conclusions:**

Carers' expectations of support from general practice for themselves are low and teams are seen as reactive and time constrained. Both the carers and the general practice team participants emphasised the valuable role of general practice team in supporting stroke survivors. Research is needed to determine general practice teams' awareness and identification of carers and of the difficulties they encounter supporting stroke carers. Carer policy initiatives need greater specificity with greater attention to diversity in carer needs.

## Background

### Informal carers and stroke survivors

Awareness of the numbers of informal carers and the important role they play is growing. It has been estimated that they save the economy £119 billion annually - more than the annual cost of the NHS [1].

Informal carers, also known as carers or caregivers, are defined as:

*'... someone who, without payment, provides help and support to a partner, child, relative, friend or neighbour, who could not manage without their help*. 'Princess Royal Trust for Carers (2009) [2]

Approximately one in ten of the population of England and Wales are carers with three in five people becoming carers at some point. Numbers are expected to increase with demographic changes [3].

In England there are estimated to be over 900,000 stroke survivors and approximately half are dependent on others for everyday activities [4]. This support frequently comes from spouses, families and friends. Informal care costs (the costs to patients and carers of paying professional carers and the time spent by the carers costed at the national mean average hourly rate) are estimated to be £2.5 billion a year.'[5].

Carers play a very important role in supporting stroke survivors [6-8] but research consistently reports adverse impacts on carers. These include negative effects on mental health, burden and stress [9,10] thus making it important they are supported both for themselves and those they care for. Numbers of stroke survivors and therefore also their carers are expected to increase as the population ages and as therapeutic interventions improve survival [11]. Understanding how best to support carers is therefore a priority.

### The role of general practice and stroke carers

General practice is often the first point of contact for carers and is well-positioned to recognise and support them [12] but despite this there is little recent published research in the area. Over a decade ago Brotheridge et al. (1998) [13] reported that both stroke patients and carers thought that their general practitioner had an important role to play and should have regular contact with them. Only carers said they wanted information and advice. Compared to the stroke survivors, carers seemed to expect more from their general practitioner and the authors suggested that carers' needs remained neglected.

In a slightly later study, Simon and Kendrick (2001) [14] found that GPs and primary care teams believed they had an important role but lacked time, resources and training. GPs tended to see themselves as reactive as opposed to proactive.

The importance of primary care in supporting stroke survivors and their carers has been highlighted [15] but research asking stroke carers specifically what they would like is rare. Hare et al. (2005) [16] used focus groups with stroke survivors and carers to determine their long-term support needs. Three themes emerged: emotional and psychological problems; lack of information and the importance of primary care as the first point of contact. However, it is not possible to identify the carers' support needs because survivor and carer perspectives were combined. A review of qualitative literature in stroke highlighted the desire for information and reported that carers wanted information about the long-term consequences of stroke and community services [17].

### Government policy and carers in general

In the UK the vital role played by carers is now being acknowledged and recognition and support for carers has been pledged [18,19]. In 2007 the New Deal for Carers was announced [20]. Aimed at carers in general, it included three practical measures to support carers based on what carers want [21]:

• A telephone helpline providing detailed, up-to-date information ranging from national rights and entitlements to what is available in carers' own areas.

• Emergency support providing short-term, home-based respite.

• A Caring with Confidence scheme to train carers to take greater control over their health and their cared-for. It includes advocacy skills and practical instruction in e.g. first aid, moving and handling.

Concern has been raised that this policy does not represent carers' perspectives. When drafting this policy the public consultation was conducted electronically over a short time period. As a result, some specific groups, such as stroke carers and older carers, may not have responded and might not find the practical proposals helpful [22].

## Aims

Firstly, this study explores both the support stroke carers would like from general practice and their reactions to the community based support proposed in the New Deal. Secondly, perceptions of a general practice team are investigated covering similar topics to carer interviews but from the practice team's perspective.

## Methods

### Interviews and topic guides

Semi-structured, face-to-face interviews were conducted with carers and the general practice team using topic guides. These guides (See Additional file 1 for the carers' topic guide) were very similar for the two groups and focussed on perceptions of what carers would like from general practice in general. Opinions of a helpline, home-based respite and carer training as provided in the New Deal (2007) [21] were also explored. This semi-structured qualitative approach gave the participants the opportunity to reflect on specific available services but also to discuss other options more widely. Interviews were recorded and transcribed verbatim.

### Carer recruitment and interviews

Carers were purposively sampled from an acute stroke unit, a rehabilitation centre and a general practice to provide a sample of carers who had been caring for a range of lengths of time. All recruitment sites were in South West London. Carers were sampled to provide a range of genders and relationships between carers and survivors (e.g. spouses and daughters). Carers, defined as the person providing the most unpaid care for a stroke survivor, could be living with or separately from survivors. All survivors were living in the community. Carer interviews were carried out by a researcher with experience in interviewing carer participants. She introduced herself to participants as a researcher, who was not a clinician and not associated with service provision.

Clinical staff in the stroke and rehabilitation units identified possible carer participants close to discharge. A researcher approached carers whilst they were on the wards, described the study and asked whether they might consider participating after discharge. It was stressed that there was no obligation to participate and that refusal would not influence their survivor's care. Carers were provided with written information and with their permission, the researcher contacted them later to determine if they were interested in participating. Following informed consent, interviews were carried out at a time and place convenient for the carers.

Carers from general practice were identified by the GP researcher (WF) from practice records of stroke survivors with known carers. He wrote to these survivors and briefly described the study. Stroke survivors were asked to pass a letter onto their carer inviting them to participate in the study. This invitation contained the same information about the study as for participants from inpatient settings and included a stamped addressed envelope to reply directly to the research team. Again it was stressed that they were under no obligation to take part and that refusal would not influence the care of either the stroke survivor or the carer. Carers who responded to the invitation were contacted and interviewed following informed consent.

### General practice team recruitment and interviews

General practice team members were recruited from one general practice. The GP researcher described the study and provided written information to practice team members. Ten team members were purposively sampled to include representatives of the professions in the team (GPs, nurses, administrative staff). Administrative staff were included because they were regarded as part of the general practice team and because they had direct contact with carers and patients. General practice team members were contacted and interviewed by one of two researchers (AM and RH both nurses) and interviews carried out at times and places convenient to participants.

### Data analysis

Interview transcripts were content analysed [23] by hand. Key themes were independently identified and categorised by two researchers (NG a social scientist and AM an academic nurse) both of whom have extensive experience in qualitative research and in analysing qualitative data. There was considerable similarity in the themes identified and consensus was easily achieved with discussion. Formal analysis did not begin until after data collection but since clear themes were emerging early on, recruitment stopped once there were no new themes were emerging [24].

### Ethical approval

Ethical approval for the study was gained from the local NHS Research Ethics Committee.

## Results

Thirteen carers were interviewed. All eleven carers approached directly from the stroke ward and the rehabilitation centre agreed to participate but only data from nine of these participants are included here. In one case the tape failed to record and in the other the carer had misunderstood the nature of the research and was not interviewed. From 32 letters sent out by the GP, four carers agreed to participate. Interviews lasted from 30-90 minutes.

All carers spoke English although it was not always their first language. Eight carers had been caring for the stroke survivor for more than six months and the remainder for less. Participants included eleven spouses and two daughters. Eight carers were female and four carers were in paid employment (Table 1). All ten members of the general practice team who were approached were interviewed. They included five GPs, two practice nurses, one nurse practitioner and two administrative staff.

**Table 1:** Carer characteristics

		N	%
**Gender**	Female	8	62%

	Male	5	38%

**Age**			

	< 60 years	3	23%

	> 60 years	10	77%

**Relationship **	Wife	6	46%

	Husband	5	38%

	Daughter	2	15%

			

**Living arrangements**	Cohabit	11	85%

		2	15%

			

**Paid employment**	Yes	4	31%

	No	9	69%

			

**Time caring **			

	Caring less than 6 months	5	38%

	Caring more than 6 months	8	62%

The themes identified from the transcripts are presented for the participants as a whole because overall there were no striking differences between, for example, female and male carers and those caring for less than six months and those caring for longer. On the one occasion where there appeared to be a difference related to length of time caring, this is highlighted below.

Themes, illustrated by quotes, are presented below. Carers are described after quotes by e.g. 'husband' or 'daughter' but no demographic information is provided for the general practice team as they might potentially be identified. General practice team's perceptions are described after carers' perceptions.

### Themes

Content analysis of the transcriptions identified several themes. There were some striking similarities in carers' and the general practice team's perceptions.

### Carer perceptions

#### Satisfaction with support from general practice for stroke survivors

Despite the focus of the interviews being on the support carers would like, carers tended to talk more about the support their stroke survivor had received, rather than about support for themselves. Satisfaction with services was not the focus of the research but this theme is included as it emphasises carers' perceptions of the role of general practice as centred on care of the stroke survivor. Most carers frequently spontaneously said they were happy with the care their stroke survivor had received.

*'He's kept an eye on us without sort of being on our back all the time.' *Husband

Where there were criticisms they tended to relate to continuity of care, especially from large practices and to perceived poor follow-up after the stroke. Several carers questioned whether the practice was aware their cared-for had suffered a stroke.

*'They didn't even know it *(the stroke) *had happened.' *Husband

#### General practice as a place for support

When asked, carers frequently said they would ask for help from their general practice but usually described this in the context of the stroke survivors' needs rather than their own needs.

*'I'm sure they would be supportive, let me put it this way, I think I haven't expected much support simply because they were very mild strokes and he hasn't needed any physical care as such. It's been mainly short term memory loss. .. it's mostly psychological and I haven't, directly approached the GP for assistance myself*.' Wife

#### Low expectations of support for themselves

Expectations were generally low but were not always met. Few carers had expected contact from their general practice but on reflection thought it might have been very helpful especially initially.

*'You know perhaps they could have rung up or called round but I wouldn't have expected it*.... *I think with hindsight, yes, I think I would have liked them to have rung me .... it's rather nice if your GP would actually spend 15 minutes with you, explaining exactly what happened.' *Wife

Carers were seldom receiving support aimed specifically at them rather than the stroke survivor but they often believed, if requested, it would be forthcoming. If carers wanted anything, emotional support and health checks were mentioned but support from general practice was not always wanted. Some carers would like emotional support with their caring role but did not think this was general practice's role.

*'I always regard them to do with medical matters rather than anything to do with the mind. I've got very good friends and I usually phone up and bore them for an hour with it*. ... *I just think of him as a medical man.' *Husband

#### General practices perceived as reactive - 'They don't have time'

Lack of time was often mentioned:

*'I've sort of been asked 'How are you?' and 'How are things?' but I have always sensed that time is limited. I don't know, I don't know what would be on offer if I asked, whether they have a counsellor.' *Wife

Carers often would have appreciated proactive contact.

*'... we've gone round there just to have the blood pressure checked at different times but ... they never say to us 'Well come every three months' or whatever - they don't say that..... actually I would have liked someone to have kind of put forward any suggestions or said 'Is there any help or advice you need?'' *Wife

Some felt the onus was on them to request help if it was needed rather than it being routinely offered.

*'... no nothing's forthcoming, but as I say I haven't contacted them so that's my own fault.' *Wife

Time constraints or others with more pressing needs were used as explanations for not proactively being offered support. Carers emphasised not wanting to 'bother' doctors.

*'Their surgery's always full of people children and everything - they don't have time - you know it takes time to speak to people like you are speaking now with me ..' *Wife

### Practical measures from recent policy

#### Information line

Carers were generally uncertain how personally helpful they would find a telephone information line. Typically they thought others might benefit but would probably not use it themselves. Some said they would prefer a drop in centre with face-to-face contact particularly for emotional support or where English was not a first language. However, several did comment that having access to email support in addition to a phone line might be good (although others stressed not wanting support via the internet).

*'I think it would *(be helpful) *simply because, my experience and other people's ... it is quite often difficult to get through to your GP. ... I can imagine if I was looking after someone very disabled and I might just be at the end of my tether...' *Daughter

*'Yes I think that would be a big help - not that we need it but for other people .... because I know it's a bit of a minefield - not personal experience - but people I know.' *Wife

Some expressed concern about how the helpline would operate. Carers highlighting their preferred choice of provider wanted volunteers with the right life experiences (because '*they actually care'*) rather than statutory services.

*'I mean doctors, nurses unless they've gone through these things either through bereavement with people who've had strokes whatever ..... they don't really understand - they know what the situation is and the effects but they don't understand ...... you've got to experience something.' *Wife

Carers felt such an information line could provide a variety of information and emotional support.

*'I think a helpline should provide the emotional support ... someone to moan to or possibly you feel you can pass a problem to or get direction as to how you can solve that problem...' *Husband

Some stressed the importance of availability, quick responses and personalised information.

*'Well obviously it depends what information you want - if you ask them a certain question you want a specific answer - you don't want someone going all round the world ....' *Wife

#### Emergency home-based respite

There was uncertainty of the value of emergency home respite. Carers saying they would not make use of it mostly said they would rely on their family or support networks. Having a stranger coming into their homes was regarded as potentially an issue for carers, stroke survivors and the rest of the family.

*'My children and husband take priority - my family would not want that.' *Daughter

Concern over the expertise of the person coming in to look after survivors was common.

*'Yes -I know it's very difficult these days but you need to know that the person coming in is not going to bully patients... is going to look after them and is not going to get physical or anything like that...' *Wife

However, some carers thought that it would be better than respite away from home and that it would be good to know it was available, if needed.

#### Carer training - Caring with Confidence

Of all the support measures presented to carers, carer training received the most positive response. All but one participant said it was a good idea in general but, again, many said they personally did not need it. Suggestions for types of training that could be useful included training in practical caring skills, information on available support, help with form filling, training in advocacy skills and looking after themselves. This was the only occasion where there appeared to be differences between the carers who had been caring for less than six months and those caring for longer. Newer carers were more likely than those caring for longer to say that they personally might benefit.

*'I think it would be wonderful .... especially if they teach you how to go about things and be more assertive. I think that might (if it was really well done) almost eliminate the helpline ...' *Daughter

### General practice team perceptions

#### General practice as a place for information and support

General practice team participants acknowledged that caring can be challenging and that they were well-placed to offer support, advice and counselling. Local council leaflets are available in the waiting room and their practice leaflet has contact information for e.g. the Stroke Association. When registering at the practice and completing the practice registration form, new patients are asked say if they regard themselves as carers but this appeared to be mainly so they could be contacted if necessary.

'We are probably quite well-placed in that sense because we may know both the patient and the carer.... Certainly we will quite often see carers under stress or with mental health problems ... financial difficulties, difficulties getting benefits or difficulties getting access in the home. So there is quite a bit we can do. Usually we are one of the first points of care for those sorts of problems.'

However, some members of the team suggested their role was primarily acute care and looking after stroke patients rather than carers. Although the difficulties facing carers were recognised, seeking carers out proactively was not seen as their role. Ideally carers would identify themselves as carers to the practice team without being asked. This was at least in part because communication from hospitals could be unreliable and identifying carers can be difficult.

'*I guess we could have a supportive role ... I mean we wait for people to come to us..... they may speak to us about how they are feeling or if they are struggling.'*

It was thought that carers may not approach them because they appreciate the time constraints on general practice teams.

'But very rarely does the carer come because they might feel they are a burden or something and they don't realise they can.'

There was sometimes clear ambivalence about proactively supporting carers.

'... I'm wary of the fact that I don't want to just interfere in something that I might be hindrance to. If I can help, sure... so I would like to think they can always call us if need be. But sometimes they might ... just want to speak to a non-medical person ... obviously we are always quite busy so we've got a few minutes on the phone, we steal a few minutes here and there.'

#### Signposting and referral

Referral onto other services was seen as an important part of their role with general practice teams acting as a link. Referral to social services was commonly mentioned by participants but referral to third sector organisations was not. However they generally *'wait for people to come to us'*.

*'Caring ... is demanding, it is stressful and you know everyone puts a lot of attention on the patient with the stroke and they forget about the impact both psychologically and physically. I've seen a lot of elderly women who are very proud themselves and will do everything for their partners ... but they just carry on. .. I'll try and help them, and ask them if they need any extra help, social services, meals on wheels ...*.'

'... one or two have come in and said 'I need help' but usually it's a third party like a district nurse that's come to me and said 'I've seen this patient and they are struggling a bit, can you instigate some care packages?'... But very rarely does the carer come because they might feel they are a burden or something...'

#### Awareness of carers and the issues facing them but find identifying carers difficult

General practice team participants said they were aware of the challenges facing carers but stressed difficulties in identifying carers and carers' unwillingness to take up the general practice team's time.

*'Some of them just get on with it and they don't want to burden us, they think we have enough on our plate, which I feel is really sad ... because we are here to help really. But a lot of people feel really proud, they feel that if they ask for help they are sort of letting themselves down ... it is up to the individual ...So yes, I haven't really seen a lot of people just come to me asking for help*.'

### General practice as reactive because of time constraints

The general practice team did not consider it was their role to be proactive primarily because of time constraints. Regular reviews were thought ideal by some but insufficient time both for the general practice team and other professionals meant this was unlikely.

'In an ideal world I would like obviously, carers having sort of regular reviews of carers coming in saying like every six months or so. Touching base with us to say, 'How have you been, are you coping well, do you need any extra help, would you like any more help, do you need a break?' ...but I know that can never happen because we are just too busy..'

'I have to say that primary care mainly reacts rather than being pro-active .... because we always have demands on the time...... if we were to find that somebody had gone out of hospital we wouldn't necessarily phone them up and say 'Is everything alright ?' We haven't got time really.'

### General practice team perceptions of practical measures in policy

#### Telephone helpline

Opinions of a telephone helpline were overwhelmingly positive as long as those providing the helpline had appropriate training and information. It was suggested that face-to-face contact might be preferable to some carers. The need for reassurance for carers was stressed. Several highlighted the importance of offering accessible information on benefits, financial support, services and medical conditions.

'Or maybe there's a fear of it happening again, what should you do, will the symptoms improve....? Maybe information on talking to other people that are going through the same thing, information on diet... and is there anything they can do to make them better or help them be more healthy.'

'So maybe it's just enough if you're having a bit of a panic you obviously can't phone the GP all of the time and a lot of people don't like to disturb the GP.... you've only got your ten minutes to see your GP and you might have to wait a few days ... if it's something small and you think 'Oh I don't want to bother the doctor about it'.'

Those mentioning possible providers thought it should not be run by general practice but by local third sector organisations. It was also stressed that a phone line should not simply redirect carers to general practice. This is partly because of time constraints and partly because they did not feel they were always the best equipped to offer the support.

'I think it would be independent. Primary care has got a lot on its plate.'

'There are some things which other people would do better than GPs. GPs generally don't have the details about benefits, or social services or even the sort of care that can be provided ....'

#### Emergency respite

Although general practice team participants were asked about emergency respite, they consistently answered in terms of planned respite. However, they recognised that whether planned or emergency respite there were both advantages and disadvantages in home respite and that it might be down to individual preference.

#### Carer training - Caring with Confidence

Opinions were mixed about carer training but were similar to those of carers. Those in favour tended to think training would benefit carers perhaps by helping them learn to look after themselves and increasing their confidence in their caring skills. It was suggested that carers might like training in practical issues such as diet, back care, lifting and handling and aids. Training in problems specifically associated with caring for a stroke survivor such as depression and personality changes were also highlighted. Some commented on issues surrounding provision of the scheme, such as ensuring ready accessibility. Opinions were divided about possible service providers but frequently both professional input (e.g. from therapists) and voluntary sector input was considered necessary.

'You'd want a carer that's been through it .... as opposed to a health professional going 'This is what you need to do' but then a health professional ... you feel like they know what they are talking about ....'

'.. the basic stuff about what's happened and then maybe about back care, how to mobilise safely for the relative and for themselves so they don't injure themselves, diet, what to do if this happens again. And where to go for support if you need it.... bathing, washing and if there are any aids that might help.'

## Discussion

The findings suggest that carers regard general practice primarily as a source of support for their stroke survivors, rather than for themselves. Despite the focus of the interviews being on carers and their support needs, carer participants tended to centre their descriptions of their experiences and the support they would like around the stroke survivor. Similarly the general practice team appeared to regard care of the stroke survivor, as opposed to their carers, as their main role.

The general practice team here expressed both interest in carers' issues and a desire to support them but had concerns about time constraints and whether they were always in the best position to offer support. Neither set of participants saw general practice teams as proactive both suggesting that time constraints were an issue. Carers here generally did not expect proactive support but assumed help would be forthcoming if requested. A decade ago it was reported that GPs and primary care teams believed they can have a key role to play supporting carers but lacked time, and saw themselves as reactive [14]. Our findings suggest that ten years later, carers and the team interviewed here would agree largely with this earlier research.

There are similarities here with Hare et al's (2005) [16] study which suggested carers and stroke survivors would appreciate emotional support and information. Carers in our study mentioned wanting emotional support but information did not appear an overriding concern. If they mentioned information, they emphasised the personalised nature of the information required such as their survivors' prognosis or benefit entitlements. Similarly the general practice team suggested carers might appreciate emotional support but that they might also like information about, for example, their survivor's stroke and financial support.

Although carers mostly thought that general practice was in a position to support them, expectations were low partly because general practice teams are seen to be focussed on the stroke survivor and partly because they do not always think they are recognised as carers who might want support. Emotional support and explanations of the stroke's implications would be appreciated but time constraints and others' perceived greater need meant this was not expected. Unlike some research [14,25] the general practice team did not emphasise lack of confidence and insufficient training in supporting carers but they did highlight time constraints stressing the reactive nature of their service and suggesting some support might be better offered by others. This was emphasised when discussing a telephone helpline where it was thought that information, for example, about benefits should be provided by organisations with appropriate expertise. They stressed their role in signposting and referral but noticeably this was not highlighted by carers.

It was also striking how carers often said the practical measures in the New Deal for Carers might help others but that they themselves did not require it. This may be a reflection of the generic nature of the support being offered [22] so that carers consider it might be generally useful but not specifically valuable for them. The general practice team were more positive about the value of the practical measures for carers in the New Deal but emphasised that they would not be in a position to offer such services.

This is an exploratory study but it has a number of strengths. Purposive sampling ensured that the carer participants came from a range of demographic backgrounds and lengths of time since stroke. Carers came from a variety of general practices although four came from the same practice as the general practice team participants. However, the opinions of these carers were indistinguishable from the carers from other practices. A larger study might have explored the perceptions of non-English speaking carers but in this study, none of the sample was unable speak English. Only the views of one albeit large, general practice team are expressed here possibly limiting the generalisabilty of the findings. Gaining perspectives of a wider range of practices and perhaps exploring specific roles for the different professionals in the general practice team would have added to the findings. Finally, service user involvement in developing the research and interpreting the findings may have added to the research.

## Conclusions

These findings emphasise that even within one specific medical condition, there is considerable diversity in the support carers want. The desire for individualised support catering for their differing situations is clear. Carers' expectations are low and general practice teams may find it difficult to offer proactive support despite recognising carers' support needs. General practice is valued for supporting stroke survivors but there is uncertainty about whether carers should self-identify themselves and what support should be expected from general practice. Further research is needed to determine the best ways for general practice teams to identify carers and what support they can feasibly offer. However additional funding for the charities and voluntary organisations already working with carers would make these services available to more carers. Carers here would mostly have appreciated contact soon after the stroke survivor's discharge. Given the evidence of the adverse impact of caring, proactive contact with carers early on might reduce difficulties later. Carers' champions might be well-placed to support carers whilst keeping input from GPs to a minimum [26] but research is needed to determine their impact. Funding restrictions are likely to reduce services. Already the Caring with Confidence scheme, seemingly the most popular practical support measure with participants here, is ending and is to be replaced with a training programme for GPs. This new programme is intended to raise GPs' awareness of their role and contribution to supporting carers [27] but our evidence suggests that, not only general practice teams, but also carers are unclear about what carers can expect. With the proposed changes in health and social care commissioning and with greater control in commissioning being assigned to primary care it is uncertain how services will be provided. General practice is well positioned to offer support to stroke survivors and carers [12] and the socioeconomic stakes are high if these carers are not supported. However, perhaps general practice is better placed to support stroke survivors and other third sector agencies may, in fact, be better equipped to support carers.

## Competing interests

The authors declare that they have no competing interests.

## Authors' contributions

All authors contributed to the study design. NG was primarily responsible for the study design, data analysis and drafting the article. WF and GC helped with participant recruitment. RH and AM interviewed the general practice staff. AM also analysed the data. All authors read and approved the final manuscript.

## Pre-publication history

The pre-publication history for this paper can be accessed here:

http://www.biomedcentral.com/1471-2296/12/57/prepub

## Supplementary Material

Additional file 1 **Carers' interview topic guide**. Carers interviews covered the topics in this guide. Click here for file
