# Supplementary material for: Perceptions of the role of general practice and practical support measures for carers of stroke survivors: a qualitative study
Source: BMC Fam Pract. 2011 Jun 23;12:57. doi: 10.1186/1471-2296-12-57 (PMC3141511; doi:10.1186/1471-2296-12-57)
Supplement: Additional file 1 — Carers' interview topic guide. Carers interviews covered the topics in this guide. [file 1471-2296-12-57-S1.DOC]

**Topic guide: Carer interviews**

**1. Expectations of primary care**

- What support if any are they receiving from primary care?
- What are/were their expectations of support?
- What would be their ideal form of support?

**2. Carers’ reactions to the practical measures in New Deal for Carers**

The New Deal for Carers pledges three main reforms to support carers in general.

The main elements (a-c) are described to participants

1. *An information service/helpline for carers, perhaps run by a voluntary organisation which would provide the right, detailed up-to-date information to assist them in their caring role. This information is intended to help them make decisions about their personal support, opportunities and the needs of the person for whom they care.*
   - How useful would this be?
   - Can they think of a time when they would have used such a service, or not?
   - Can they provide examples of the sorts of information they would like?
   - How could it best be set up?
   - How would they like it to be administered?
2. *Emergency support is to be established in each local authority area providing short-term, home based, respite for carers in crisis or emergency situations.*
   - How useful would this be?
   - Can they think of a time when they would have used such a service, or not?
   - How could it best be set up?
3. *Expert carers programme – this will be aimed at providing training for carers to develop the skills needed to take greater control over their own health and the health of those in their care.*
   - How useful would this be?
   - What training would carers want?
   - How could it best be set up?
   - Would they personally value such training?
